# Investigating vesicle-mediated regulation of pollen tube growth through BFA inhibition and AS-ODN targeting of *TfRABA4D* in *Torenia fournieri*

**DOI:** 10.1093/hr/uhaf018

**Published:** 2025-01-15

**Authors:** Xingyue Jin, Akane G Mizukami, Satohiro Okuda, Tetsuya Higashiyama

**Affiliations:** Department of Biological Sciences, Graduate School of Science, The University of Tokyo, Faculty of Science Building 2, The University of Tokyo, Hongo Campus 7-3-1 Hongo, Bunkyo-ku, Tokyo 113-0033, Japan; Department of Biological Sciences, Graduate School of Science, The University of Tokyo, Faculty of Science Building 2, The University of Tokyo, Hongo Campus 7-3-1 Hongo, Bunkyo-ku, Tokyo 113-0033, Japan; Division of Liberal Arts and Sciences, Aichi Gakuin University, 1-100 Kusumoto, Nisshin, Japan; Department of Biological Sciences, Graduate School of Science, The University of Tokyo, Faculty of Science Building 2, The University of Tokyo, Hongo Campus 7-3-1 Hongo, Bunkyo-ku, Tokyo 113-0033, Japan; Department of Biological Sciences, Graduate School of Science, The University of Tokyo, Faculty of Science Building 2, The University of Tokyo, Hongo Campus 7-3-1 Hongo, Bunkyo-ku, Tokyo 113-0033, Japan

## Abstract

In flowering plants, pollen tube growth is essential for delivering immotile sperm cells during double fertilization, directly influencing seed yield. This process relies on vesicle-mediated trafficking to sustain tip growth and fertility. However, investigating pollen tube growth is challenging in non-model plants due to the lack of transgenic tools. Here, we developed a method to transiently inhibit vesicle activity in pollen tubes of the wishbone flower (*Torenia fournieri*), a classic plant for sexual reproduction studies, using brefeldin A (BFA) and antisense oligodeoxynucleotides (AS-ODNs) targeting key genes. BFA broadly disrupted vesicle gradient homeostasis in *T. fournieri* pollen tubes, leading to widespread changes in cell wall deposition, ROS distribution, and pollen tube morphology. To assess the role of specific genes, we designed AS-ODNs against *TfANX*, the sole *ANXUR* homolog in *T. fournieri*, which successfully penetrated cell membranes and suppressed *TfANX* expression. This inhibition impaired pollen tube tip growth, causing pollen tube leakage at the shank region and, in some cases, multiple leakages. Similarly, AS-ODN targeting *TfRABA4D*, a pollen-specific vesicle regulator, induced a bulging phenotype and disrupted pectin deposition and reduced ROS distribution, mirroring BFA effects. These findings elucidate vesicle-mediated regulation in pollen tube tip growth and introduce an accessible method for genetic manipulation in reproductive research of non-model plants.

## Introduction

The delivery of sperm cells via tip-growing pollen tubes is a unique feature of seed plants, and requires precise regulation of tube growth during reproduction [1]. The pollen tubes of flowering plants exhibit rapid growth rates, e.g. ≤ 2.8 μm∙s^−1^ in maize (*Zea mays*) and 0.2–0.3 μm∙s^−1^ in lily (*Lilium longiflorum*), necessitating a sophisticated vesicular trafficking system to transport secretory materials to the plasma membrane of the pollen tube tip, as well as coordination between endocytosis and exocytosis at the tip [2–4]. Defects in tip growth are a primary cause of low fertility in artificially bred plant species [5,6]. Thus, investigating the functions and mechanisms of vesicle transport in pollen tubes can provide insight into reproductive processes in angiosperms and facilitate the development of novel plant-breeding strategies.

Vesicle-mediated membrane trafficking, including exocytosis and endocytosis, is crucial for plant growth and reproduction [7]. In some plant taxa, such as *Arabidopsis thaliana*, maize, and tobacco (*Nicotiana tabacum*), the factors controlling vesicle trafficking have been investigated [8–10]. For instance, SNARE proteins, which mediate vesicle fusion with the target membranes, classified into R-SNAREs (contributing an arginine [R] residue) and Q-SNAREs (contributing a glutamine [Q] residue), interact to assemble a tetrameric trans-SNARE complex and regulate vesicle trafficking [11, 12]. YKT61 is an R-SNARE protein in *A*. *thaliana*. Mutant *ykt61–3* does not interact with multiple SNARE proteins and shows substantially reduced membrane association and male infertility [13, 14]. SYP121, a maize Q-SNARE protein localized to the plasma membrane, regulates K^+^ channel trafficking and cellular osmotic homeostasis [15, 16]. Rab GTPases, such as RabA2, directly bind to SNARE proteins, coordinating vesicle budding and fusion in pollen tubes of *A*. *thaliana* and tobacco [9, 17–19]. Genetic interference with *RabA4d*, the sole member of the *RabA4* subfamily expressed in pollen, leads to disturbances in wall material deposition and the formation of shorter and wider pollen tubes in *A*. *thaliana* [19]. The functional loss of NtRab11b, a homolog of *A*. *thaliana* RabA1s, disrupts the directional growth of pollen tubes due to abnormal vesicle distribution and secretion in tobacco [9]. However, severe mutant phenotypes, such as failure to develop or germinate, abnormal tube elongation, or affected transmission of the male gametes, hinder the generation of stable progeny [20], emphasizing the need for alternative experimental systems that allow the investigation of key genes involved in pollen tube regulation in non-model plants.

Chemical inhibitors, which have high specificity, reversibility, and rapid efficacy, enable the investigation of biochemical events in plants [21]. For example, the fungal macrocyclic lactone brefeldin A (BFA) disrupts vesicle trafficking by inhibiting the activity of an ADP ribosylation factor guanine nucleotide exchange factor [22]. On this basis, BFA has been used to investigate the secretory pathway in pollen tubes. In lily and conifer, BFA arrests pollen tube growth by disrupting vesicle trafficking and impeding the secretion of cell wall material, ultimately affecting growth polarity [23, 24]. Antisense oligodeoxynucleotides (AS-ODNs) can be used to selectively inhibit gene expression. For instance, in pear, *in vitro* pollen tube growth is hindered, and reactive oxygen species (ROS) accumulation is promoted by AS-ODNs targeting *periwinkle* (*Catharanthus roseus*) *receptor protein kinase PbrCrRLK1L13* [25]. AS-ODNs specifically inhibit the expression of *NtGNL1* in tobacco pollen tubes, leading to abnormalities in pollen tube endocytosis [26]. Indeed, in *A*. *thaliana* pollen tubes, phosphorothioate AS-ODNs targeting *ANX*, *CalS5*, and *ROP1* leads to growth defects, aiding the identification of genes that play an important role in reproduction [20]. Therefore, the effects of chemicals on pollen tube growth provide a useful approach for studying genes that affect fertility, up to and including causing infertility [27].

**Figure 1:**
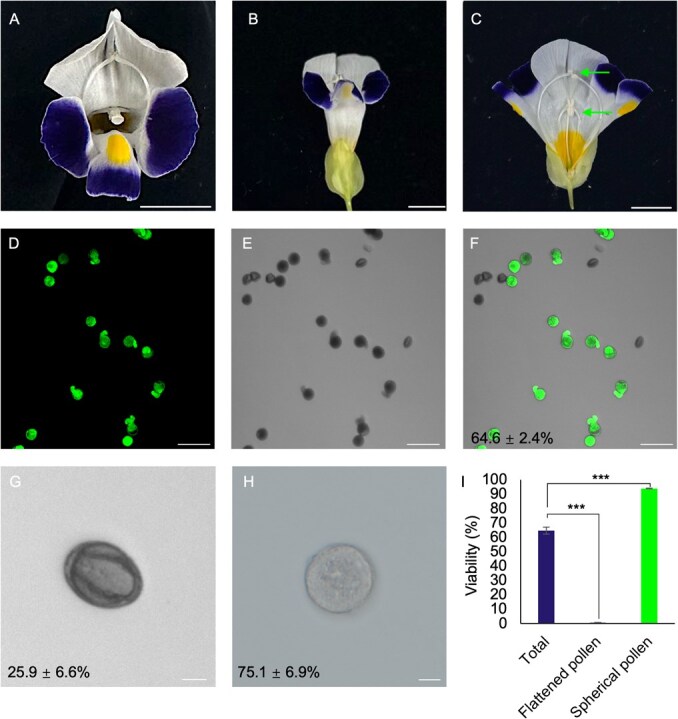
*Torenia fournieri* flower morphology and pollen viability analysis. (**A–C**) Top view (**A**), front view (**B**), and opened view (**C**) of a *T. fournieri* flower 2 days after anthesis. The arrows (upper and lower) indicate the upper anthers and bottom anthers, respectively. Scale bars: 1 cm. (**D–F**) Pollen grains from the bottom anthers stained with FDA (**D**), under the bright field (**E**), and merged image (**F**). Scale bars: 100 μm. (**G**, **H**) Morphology of flattened (**G**) and spherical (H) pollen grains. Scale bars: 10 μm. Values of pollen viability (**F** and **I**) and pollen proportion (**G** and **H**) are means and standard deviations of more than 3 replicate experiments (*n* > 100). ^***^P < 0.001 from the total.

The wishbone flower (*Torenia fournieri*), an annual flowering plant, serves as a model for research on the reproduction of horticultural plants because its naked embryo sac enables the investigation of essential fertilization events [28, 29]. Ovular attractant peptides (LUREs) and the bioactive arabinogalactan sugar chain (AMOR) are important for pollen tube guidance in *T*. *fournieri* [30–32]. Pollen tubes of *T*. *fournieri* can be studied using microfluidics [33]. However, due to the lack of easily accessible transgenic methods, reproductive research in this unique plant has been limited. Here, we applied BFA and AS-ODN to *T*. *fournieri* to assess the function of vesicle trafficking in pollen tube growth and its association with cell wall formation through cytochemical labeling, immunostaining, and live imaging.

BFA disrupted the distribution of vesicles in the clear zone and affected the deposition of cell wall materials, affecting tip growth. AS-ODNs entered pollen tubes in a vesicle-dependent manner and downregulated target genes, for which *ANX* was used as a marker. In addition, targeted downregulation of *TfRabA4d* by AS-ODN resulted in a shortened and bulging pollen tube with altered ROS distribution, an effect similar to BFA, implicating RabA4d in vesicle trafficking and ROS accumulation. In conclusion, we used cytochemical, immunofluorescence, and molecular biological techniques to establish the AS-ODN technique in *T*. *fournieri* and characterized the functions of key genes in vesicle trafficking during pollen tube tip growth. The method enables manipulation of the growth of the pollen tube tip, thereby paving the way for future crop enhancement.

## Results

### Inhibition by BFA disrupts vesicle distribution and pollen tube growth

*Torenia fournieri* has two pairs of anthers, with the bottom pair opening later and containing fresher pollen 2 days after anthesis (Fig. 1). The bottom anthers produce spherical (75.1 ± 6.9%) and flattened (25.9 ± 6.6%) pollen grains. FDA staining revealed high viability in spherical grains (93.9 ± 0.3%) but almost none in flattened grains (0.6 ± 0.0%). Therefore, only spherical grains from the bottom anthers were used for subsequent studies.

**Figure 2:**
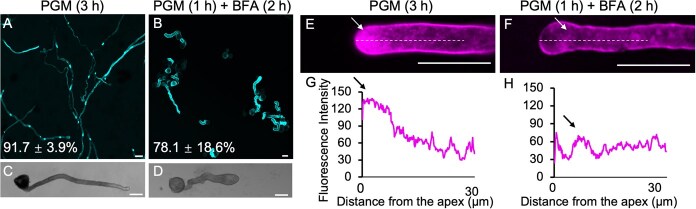
Effect of BFA on pollen germination, pollen tube morphology, and vesicle distribution in *T. fournieri*. (**A**) Pollen tubes cultured in standard PGM for 3 h showed a germination rate of 91.7 ± 3.9% and a normal shape (*n* > 200). (**B**) Pollen tubes cultured in PGM for 1 h followed by PGM containing 4 μg/ml BFA for 2 h showed a germination rate of 78.1 ± 18.6% and bulging (*n* > 200). The pollen tubes in A and B were stained with Aniline blue. Values of the germination rate are means and standard deviations. (**C**, **D**) Typical morphology of pollen tubes cultured in (**A**) and in (**B**). (**E**, **F**) Vesicle distribution in the pollen tube stained with FM4–64 in PGM (3 h) and in PGM (1 h) + BFA (2 h). Scale bars: 20 μm. (**G**, **H**) Relative fluorescence intensity on the white dashed line in **E** and in **F**. Arrows indicate accumulations of secretory vesicles in the apical zone (**E**, **F**); the corresponding fluorescence intensities are shown in (**G**, **H**). Accumulated vesicles in **F** (arrow) formed the BFA compartment.

To investigate the role of vesicle transport in *T*. *fournieri* pollen tubes, we disrupted vesicle activity using BFA. Pollen was germinated in pollen germination medium (PGM) containing 1, 2, 3, or 4 μg/ml BFA, based on previous reports (0.1–20 μg/ml in tobacco [24, 34], 0.1 to 5 μg/ml in *A*. *thaliana* [23, 35, 36], 1 μg/ml in lily [23], and 8 μg/ml in a conifer [24]), with 4 μg/ml identified as the most effective concentration for inhibiting vesicle trafficking in *T*. *fournieri* (Fig. S1). After germinating for 1 hour in PGM, pollen tubes were treated with 4 μg/ml BFA for 2 hours (PGM 1 h + BFA 2 h) to assess vesicle function (Fig. S1). The BFA treatment resulted in slowed pollen tube growth with morphological abnormalities. Specifically, the germination rate decreased to 78.1 ± 18.6%, compared to 91.7 ± 3.9% in the PGM control, indicating that late-stage germination was inhibited. In addition, the tips of BFA-treated pollen tubes showed a shorter and bulging phenotype (Fig. 2A–D).

To determine whether the reduced growth of pollen tubes was due to inhibition of vesicle trafficking, we used the amphiphilic dye FM4–64 to label vesicles in living pollen tubes of *T*. *fournieri*. FM4–64 consistently labeled the cell membrane and vesicles of normally growing pollen tubes, with intense staining in the apical region (Fig. 2E and G). BFA-treated pollen tubes had a wider distribution of signal, but intense apical staining was absent, and the total signal intensity (represented by the area under the curve) was approximately 50% of untreated pollen tubes. The signals aggregated at the subapical region of BFA-treated pollen tubes, a location referred to as the BFA compartment (Fig. 2F and H). Live imaging further showed a correlation between vesicle apical accumulation and pollen tube tip growth (Movies S1,2).

### Disruption of vesicle distribution influences the composition of the pollen tube cell wall and reduces ROS accumulation

**Figure 3:**
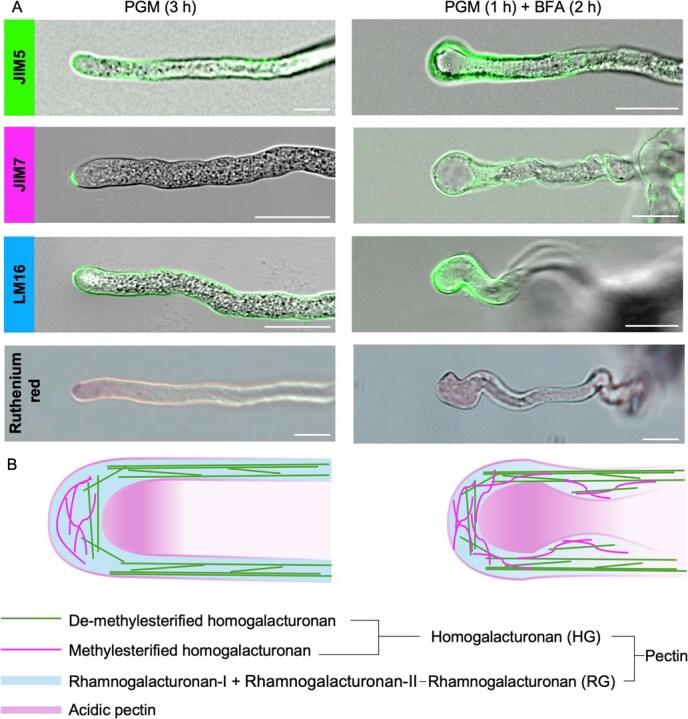
Pectin labeling in pollen tubes in the presence and absence of BFA. (**A**) Distribution of pectin components in pollen tubes cultured on PGM (3 h) or PGM (1 h) followed by BFA (2 h), labeled using JIM5, JIM7, and LM16 monoclonal antibodies, and stained with Ruthenium red. Scale bars: 20 μm. (**B**) Schematic representation showing the distribution of various pectin components. Each treatment had three biological replicates.

The pollen tube cell wall, crucial for rapid tip growth, consists of an inner layer of callose (β-1,3 glucan) and an outer fibrillar layer predominantly composed of pectin, including rhamnogalacturonan (RG) and homogalacturonan (HG) [37]. RG-I, a subtype of RG, is detectable with the LM16 antibody, while HG exists in both methylesterified and de-methylesterified forms, identified by the JIM7 and JIM5 antibodies, respectively [37]. De-methylesterified HG, visualized using the JIM7 antibody, contributes to the cell wall's flexibility and structural integrity. Ruthenium red is a general dye for pectin with a broad range of acidic pectin, typically visualizing the tip-focused accumulation of pectin in pollen tubes (Fig. 3).

Immunostaining with JIM5, JIM7, and LM16 antibodies revealed that de-methylesterified HG (detected by JIM5) is present at the apex even during normal growth in *T. fournieri*, indicating early de-methylation, as also confirmed by LM19, another antibody that specifically detects de-methylesterified HG (Fig. 3, Fig. S2). BFA treatment caused both methylesterified and de-methylesterified HG to exhibit similar distribution patterns, disrupting the pectin gradient. Additionally, LM16 and Ruthenium red staining showed increased accumulation and intensity at the swollen tips of BFA-treated pollen tubes (Fig. 3A). These results underscore the dynamic nature of pectin modification and distribution in *T. fournieri* pollen tubes, and also demonstrate that BFA disrupts vesicle transport, altering the crosspoint of methylesterified and de-methylesterified HG and thus affecting tube morphology. Additionally, cytochemical labeling with CellMask™ showed no notable differences in the cell membrane structure between control and BFA-treated tubes, while Aniline blue staining revealed significant callose aggregation after 1 to 2 hours of BFA treatment (Fig. S3). These findings suggest that BFA-induced disruptions in vesicle transport impair cell wall modification, resulting in the swollen morphology of pollen tubes.

**Figure 4:**
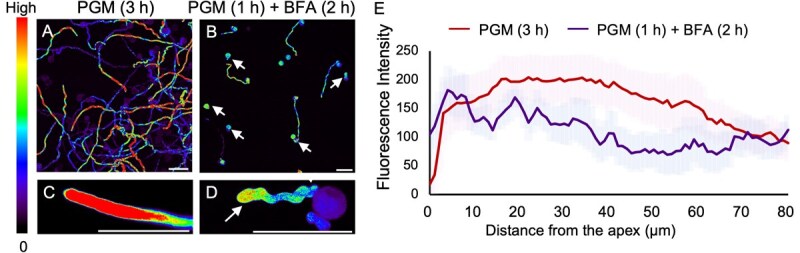
Effect of BFA treatment on ROS distribution in pollen tubes of *T. fournieri.* (**A, B**) Images of ROS distribution in pollen grains germinated in PGM (**A**) and treated with BFA (**B**). Scale bars: 50 μm. (**C, D**) Representative images of ROS distribution in the pollen tube tips of **C** and **D**. Scale bars: 50 μm. Arrows indicate abnormally enlarged pollen tubes. (**E**) Quantification of DCF fluorescence intensities along pollen tubes in PGM and BFA treatment conditions. Solid lines indicate the average fluorescence intensity of pollen tubes, with shaded regions representing the standard error (SE) calculated from three independent experiments (*n* = 5).

Tip-localized reactive oxygen species (ROS) act as signaling molecules and enhancing cell wall stability by activating pectin methylesterases (PMEs) [38]. To investigate whether this abnormal pectin gradient is also regulated by ROS, we examined ROS distribution in *T. fournieri* pollen tubes, first. In untreated tubes, ROS levels were high at the apex, gradually decreasing along the tube length (Fig. 4C). In contrast, BFA treatment significantly reduced ROS levels at the tip, disrupting apical ROS polarity and leading to a more diffuse distribution (Fig. 4D and E). This suggests that BFA does not enhance ROS production but rather impedes its accumulation. Although direct evidence of BFA's effect on ROS is lacking, similar disruptions in ROS localization and pollen tube growth have been observed in *Arabidopsis abcg28* mutants, as impaired vesicle trafficking disrupts tip-localized ROS, affecting pollen tube growth and resulting in sterility [39]. These findings suggest that BFA's interference with vesicle trafficking may similarly impact ROS localization and, thereby influencing pollen tube tip growth.

### ODN enters the pollen tube from the tip in a vesicle-dependent manner

**Figure 5:**
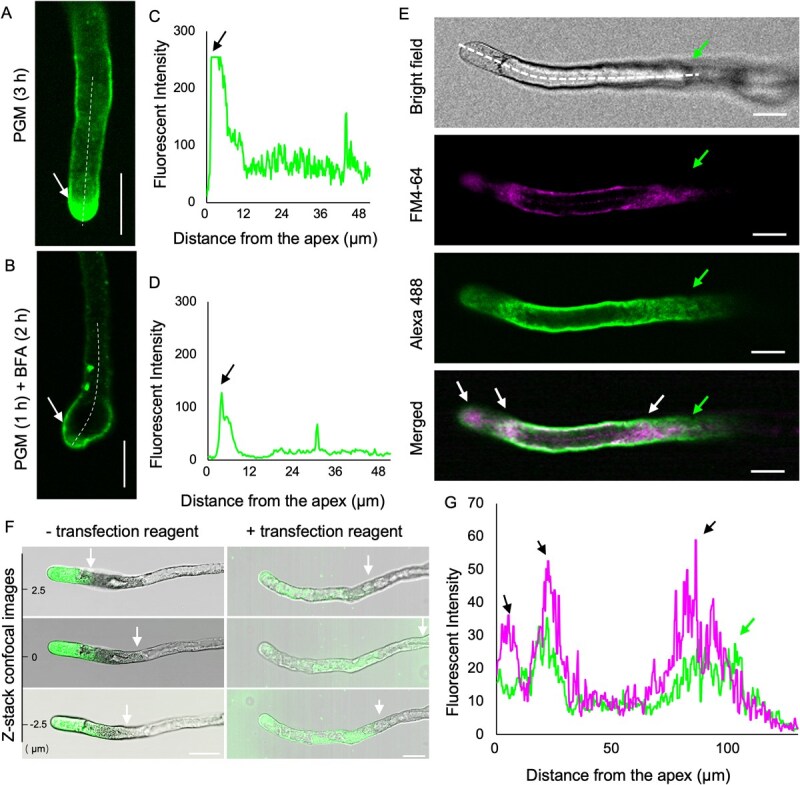
Uptake of AS-ODNs into pollen tubes. (**A**) Alexa488-labeled ODN entering the tip of the pollen tube cultured on PGM (3 h). Scale bars: 10 μm. (**B**) Alexa488-labeled ODN entering the tip of the pollen tube cultured on PGM (1 h) and BFA (2 h). Scale bars: 10 μm. (**C**, **D**) Fluorescence intensities of **A** and **B**; arrows indicate clear zones. (**E**) Pulse-chasing labeling with Alexa 488-labeled ODNs and FM4–64 in the same pollen tube. Scale bars: 10 μm. (**F**) Z-stack confocal images acquired at 2.5-μm intervals from the top to the bottom of the pollen tube. The middle three sections were selected to illustrate the entry of Alexa488-labeled ODN into the pollen tube with and without transfection reagent. Arrows indicate the farthest location Alexa 488-labeled ODN signals detected. Scale bars: 10 μm. (**G**) Fluorescence intensities of **E**; Arrows indicate clear zones and the start and end points of the vacuole. The rightmost arrows indicate the point of Alexa 488-labeled ODN signals located posterior to the vacuole. Each treatment had three biological replicates.

AS-ODNs have been employed to modulate gene expression in pollen tubes in tobacco and *A*. *thaliana*, inspiring us to explore their potential use in *T. fournieri* [20, 26]. We first ensured that AS-ODNs could pass through the pollen tube cell wall and plasma membrane. *TfANX*-targeting ODNs labeled with AlexaFluor488 were used to visualize whether ODNs were incorporated into the pollen tubes germinated on PGM. After ~1 hour, the Alexa 488 signal accumulated at the pollen tube tip, and small punctate Alexa 488 signals were evident in the cytoplasm (Fig. S4). After 3 hours, the signal was widely distributed throughout the pollen tube, indicating the penetration of ODNs (Fig. 5A and C). It has been reported that ODNs may enter pollen tubes in a vesicle-dependent manner [26]. To determine if this was the case in *T*. *fournieri*, we visualized the uptake of Alexa 488-labeled ODNs in pollen tubes cultivated in PGM (1 hour) and BFA (2 hours). The Alexa488 signal notably decreased in pollen tubes cultivated in PGM (1 hour) and BFA (2 hours), suggesting that the inhibition of vesicle activity by BFA affected the uptake of ODNs (Fig. 5B and D).

ODN uptake occurs first at the tip and then throughout the pollen tube is similar to, but slower than, FM4–64 uptake. We also investigated whether ODNs entered *T*. *fournieri* pollen tubes by vesicle-mediated endocytosis. The Alexa 488-labeled ODN and FM4–64 (to visualize endocytosis) signals overlapped at the tip (Fig. 5E) but gradually decreased in intensity and the colocalization was disrupted in the shank. The FM4–64 signal was significantly stronger than the Alexa 488-labeled ODN signal in the large vacuolar region (Fig. 5E). The Alexa 488-labeled ODN signal did not completely overlap with that of FM4–64, which implies that some ODN translocates into the cytoplasm.

In addition, prior research has indicated that elevated sucrose concentrations in the culture medium can enhance ODN uptake [26]. In this study, media with sucrose concentrations of 0, 10, 30, 60, and 90 mM were evaluated. It was observed that in PGM with 0 mM sucrose, there was almost no uptake of ODNs, while with increasing sucrose concentrations, fluorescence indicative of ODN uptake in the pollen tube gradually increased, indicating that sucrose indeed plays a role in ODN uptake (Fig. S5). Considering both the pollen germination rate and pollen tube growth rate, PGM with 30 mM sucrose was consistently used for culturing *T. fournieri* pollen tubes and performing ODNs' manipulation in this study.

To further illustrate the entry of ODNs, Z-stack confocal imaging was performed at 2.5-μm intervals from the top to the bottom of the pollen tube, with the middle three sections analyzed (Fig. 5F). This imaging confirmed that Alexa488-labeled ODNs were distributed within the intracellular compartment of pollen tube cells, demonstrating their ability to access intricate cellular structures independently. Notably, the use of a water-soluble cationic polymer-type transfection reagent, which binds to ODNs to form cationic complexes, significantly enhances this process [40]. These complexes interact with the negatively charged cell membrane, facilitating endocytosis, and once inside the cell, the reagent releases the ODNs into the cytoplasm [51]. Importantly, the use of this reagent did not affect the germination rate of *T. fournieri* pollen (Fig. S1). Consequently, subsequent target gene studies in this research employed ODNs delivered as cationic polymer complexes with transfection reagents to ensure more efficient entry into pollen tube cells.

### Inhibition of *TfANX* expression by AS-ODN alters pollen tube growth *in vitro*

**Figure 6:**
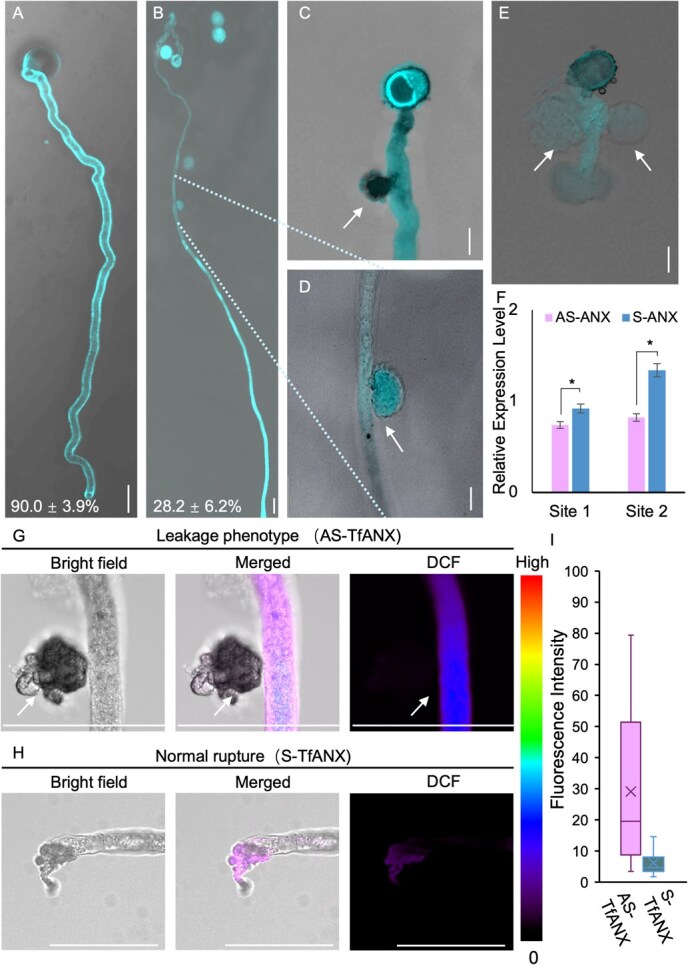
Inhibition of pollen tube growth by AS-ODNs to *TfANX in vitro.* (**A**) Typical phenotype of elongated pollen tubes germinated under S-TfANX treatment. (**B**) Specific phenotype of elongated pollen tubes under AS-TfANX treatment. Values in (**A**) and (**B**) represent the percentage of pollen tubes exhibiting the shown phenotype among the germinated pollen tubes. Values are means and standard deviations of more than three replicate experiments (*n* > 200). (**C**) Early leakage occurring in the basal region under AS-TfANX treatment. (**D**) Early leakage occurring in the middle region under AS-TfANX treatment, magnified from **B**. (**E**) Multiple early leakages under AS-TfANX treatment. The pollen tubes in **A–E** were stained with Aniline blue. Scale bars: 20 μm. (**F**) The relative expression level of *TfANX* in AS-ANX- and S-ANX-treated pollen tubes relative to the *GAPDH* gene. Site 1 indicates that the qRT-PCR primers were designed to span the ODN target site, while Site 2 primers were located downstream of the ODN site. Sequence details are available in Table S1. * P < 0.05 from the total. The assay results were generated with three technical replicates. (**G**, **H**) Images ROS distribution of pollen grains germinated in 10 μM AS-TfANX (**G**) with live imaging presented in Movie S3 and 10 μM S-TfRABA4D (**H**) medium. Scale bars: 50 μm. The ROS distribution was detected with CM-H2DCFDA. (**I**) Fluorescence intensity of DCF in the 50 μM range at the Leakage/Rupture site of **G** and **B**.

To assess the effect of AS-ODN on target genes in *T*. *fournieri* pollen tubes, we designed an AS-ODN and its sense control (S-ODN) targeting *TfANX* based on the conserved region shared by *ANX1* and *ANX2*, which are implicated in the maintenance of pollen tube growth (Fig. S6). The addition of ODNs to PGM affected the pollen germination rate in a concentration-dependent manner (Fig. 6, Fig. S7). At 1 μM, neither AS-ODN nor S-ODN significantly affected the pollen germination rate or pollen tube morphology. There were slight differences in germination rates with the application of 5 μM ODNs, but ODNs at 10 μM were optimal, resulting in more pronounced phenotypes (Fig. S7). Therefore, we used 10 μM ODNs in subsequent experiments, which was lower than that used for *A*. *thaliana* (20 μM) [ 38]. Treatment of pollen with 10 μM ODNs significantly reduced *TfANX* expression in pollen tubes (Fig. 6F), indicating that AS-ODN is capable of modulating gene expression in *T*. *fournieri* pollen tubes *in vitro*. Furthermore, AS-ODN caused leakage (28.2 ± 6.2%) of pollen tubes at random locations, including the basal region, middle region, and even multiple sites, resembling the phenotypes observed in *A*. *thaliana anx1/2* mutant [41]. Therefore, it is indicated that this method can be effectively used to validate gene function, particularly in cases where generating knockdown or knockout mutants is challenging.

The leakage phenotype induced by *TfANX* knockdown exhibits significant differences in both the location and morphology of the released contents compared to typical rupture events. In most plants, localized ROS concentrations notably increase during pollen tube rupture to release sperm cells. To explore whether ROS is involved in these AS-TfANX–induced leakages, we measured ROS levels (Fig. 6G–I). Interestingly, while normal rupture events lead to a marked reduction in ROS distribution at the pollen tube tip, leakage events at the shank region triggered by AS-TfANX retain ROS activity at the leakage site (Fig. 6G and I). We further used live imaging to monitor dynamic ROS changes throughout multiple leakages induced by AS-TfANX treatment (Movies S3). Under identical laser conditions and exposure times, pollen tubes treated with S-TfANX showed no leakages, confirming that the laser was not responsible for the observed leakage or rupture [42] (Movies S4). While live imaging revealed no ROS accumulation before leaking at the site of the second leakage in AS-TfANX–treated pollen tubes, further indicating that these leakages result from compromised pollen tube integrity (Movies S3).

### Modulation of *TfRABA4D* expression by AS-ODN alters vesicle dynamics and pollen tube morphology in *T*. *fournieri*

**Figure 7:**
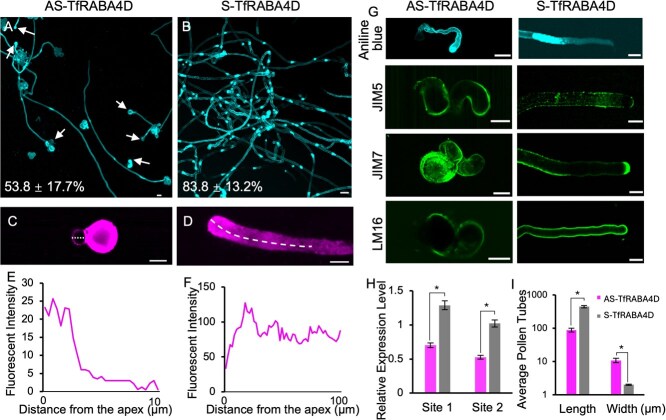
Effects of AS-ODNs to *TfRABA4D* on pollen tube morphology and cell wall patterning. (**A**, **B**) Images of pollen grains germinated in 10 μM AS-TfRABA4D (**A**), and 10 μM S-TfRABA4D medium (**B**). Scale bars: 20 μm. The pollen tubes in A and B were stained with Aniline blue. Arrowheads indicate abnormally enlarged pollen tubes. Values represent the proportion of pollen tubes exhibiting shown phenotype among germinated pollen tubes. (**C**, **D**) Images of vesicle distribution by FM4–64 staining in pollen tubes in 10 μM AS-TfRABA4D (**C**), and 10 μM S-TfRABA4D medium (**D**). Scale bars: 20 μm. (**E**, **F**) Fluorescence intensities of **C** and **D**. (**G**) Callose and pectin distribution in pollen tubes treated with AS- or S-TfRABA4D and stained with Aniline blue or immunostained using JIM5, JIM7, and LM16 monoclonal antibodies. Scale bars: 20 μm. (**H**) Relative expression levels of *TfRABA4D* in AS- and S-TfRABA4D-treated pollen tubes relative to the *GAPDH* gene. Site 1 indicates that the qRT-PCR primers were designed to span the ODN target site, while Site 2 primers were located downstream of the ODN site. Sequence details are available in Table S1. *P < 0.05 from the total. (**I**) Average length and width of AS- and S-TfRABA4D-treated pollen tubes. *P < 0.05 from the total. Values are means and standard deviations of more than 3 replicate experiments (*n* > 200).

The RabA family of Rab GTPases, particularly RABA4D, plays a critical role in regulating vesicle formation, motility, and tethering, particularly in membrane trafficking linked to the trans-Golgi network [19]. To gain a detailed understanding of vesicle trafficking network, we used AS-ODN to suppress *RABA4D*'s expression. TfB078315, the closest homolog of RABA4D with high expression in pollen tubes, was selected as TfRABA4D (Fig. S8). Based on experimental results, AS-TfRABA4D-treated tubes were shortened and had bulges at the tips, and their *RABA4D* expression was reduced significantly compared to S-TfRABA4D-treated pollen tubes (Fig. 7). This phenotype resembled of pollen tubes treated with BFA, indicating that TfRABA4D modulates vesicle trafficking. The uptake of FM4–64 was significantly decreased in AS-TfRABA4D-treated tubes, confirming that TfRABA4D modulates vesicle dynamics in *T*. *fournieri* (Fig. 7C–F).

The downregulation of *TfRABA4D* also influenced the dysregulated deposition of pollen tube cell wall components (Fig. 7G). Aniline blue staining revealed a uniform callose distribution throughout AS-TfRABA4D-treated pollen tubes, compared to reduced callose deposition at the tip and spots of accumulation in sense-treated pollen tubes like the effects observed with BFA treatment. In AS-TfRABA4D-treated pollen tubes, the distribution of methylesterified HG (detected by JIM7), which typically accumulates at the tip in S-TfRABA4D-treated pollen tubes, was dispersed throughout the pollen tube. The distribution of RG-I (detected by LM16) was more diffuse compared to S-TfRABA4D-treated pollen tubes. These findings implicate *TfRABA4D* in the vesicle trafficking upon which pollen tube patterning depends.

In addition, we further investigated whether AS-TfRABA4D affects ROS distribution at the pollen tube tip. In S-TfRABA4D-treated *T. fournieri* pollen tubes, ROS accumulation at the tip is comparable to that in untreated tube. The live imaging data shows that in S-TfRABA4D-treated *T. fournier*i pollen tubes, ROS remained concentrated at the tip, but in AS-TfRABA4D-treated tubes, ROS levels were visibly reduced, with the typical tip-focused accumulation disrupted (Fig. S9, Movies S5, S6). This reduction mirrors the ROS distribution observed in the bulging pollen tube of *osmtd2–2* in rice and *rbohh-1/rbohj-2* in *Arabidopsis*, implying the critical role of localized ROS activity in maintaining pollen tube morphology across plant species [43, 44].

## Discussion

The crosstalk between molecular pathways and cellular processes within pollen tubes is essential for sustaining their tip growth, a key process in double fertilization, which in turn supports reproductive success and contributes to the genetic diversity of flowering plants [45]. Despite its fundamental importance, research on this process has been limited in non-model flowering plants. In this study, we applied BFA to germinated *T*. *fournieri* pollen tubes and conducted biochemical and immunofluorescence analyses to ascertain the effect of BFA-induced inhibition of vesicle movement on pollen tube endocytosis and exocytosis. However, chemical inhibitors are toxic to pollen tubes and have multiple physiological effects, hampering the deeper investigation of pollen tube tip growth.

Applying AS-ODNs to complementary mRNA sequences has been used to selectively suppress gene expression and investigate gene functions [20]. However, the negative charge of ODNs hampers their passage across the plant cell wall. In terms of *T. fournieri*, we observed that ODNs could successfully enter the pollen tube cells both in the presence and absence of cationic lipids, though in some cases, the invasion distance was significantly enhanced when cationic lipids were adopted (Fig. 5F) [46]. It was reported that sucrose and active transport via sugar translocators can significantly enhance AS-ODN uptake [26]. Thus, we additionally tested pollen tube ODN uptake using PGM with varied sucrose concentrations ranging from 0 mM to 90 mM, and identified PGM containing 30 mM sucrose with a cationic transfection reagent as the optimal medium for conducting AS-ODN experiments in *T. fournieri* (Fig. 5F, Fig. S5). Interestingly, BFA, which inhibits vesicle activity, reduced ODN uptake by *T. fournieri*’s pollen tubes (Fig. 5B). Moreover, the overlap between Alexa488-labeled ODNs and FM4–64 at the pollen tube tip suggested that specialized membrane trafficking at the apex facilitates AS-ODN uptake (Fig. 5E). The mechanism of ODN uptake warrants further investigation because BFA inhibits exocytosis rather than endocytosis; however, our subsequent results suggested that it is plausible that ODNs escape from endosomes to the cytosol to inhibit gene expression.

**Figure 8:**
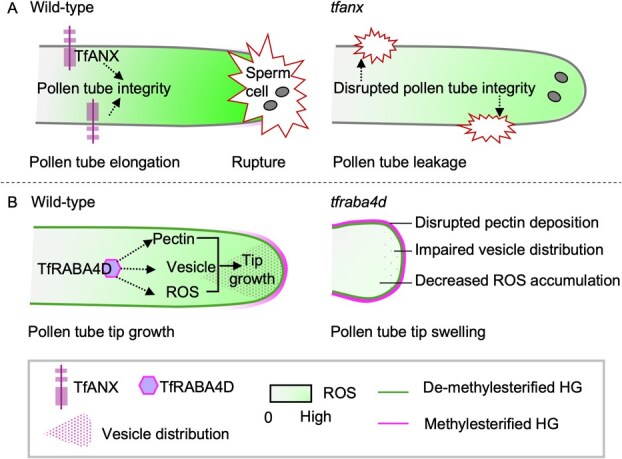
Schematic diagram of TfANX- and TfRABA4D-mediated pollen tube integrity and tip growth in *T. fournieri.* (**A**) TfANX regulates pollen tube integrity in a ROS-independent way, leading to the leakage in developing pollen tubes, distinct from rupture for sperm cell release in the mature pollen tube. (**B**) TfRABA4D supports tip growth by coordinating pectin deposition, vesicle distribution, and ROS accumulation, preventing abnormal swelling at the tip.

In pollen tubes, the specialized haploid cell responsible for tip growth relies on dynamic cytological activities [5]. However, because of their functional importance, mutations in these regulators can lead to lethality, hampering in-depth mechanistic analysis [20]. Here, in this study, AS-ODNs targeting key genes were used to knock down gene expression, resulting in *T. fournieri* pollen tubes with reduced expression of the key genes, facilitating the detailed study of molecular signaling and biological processes in *T. fournieri* pollen tubes (Fig. S10). Rab GTPases, such as Rab11b and RabA4d, localize to the tip of pollen tubes and regulate vesicle targeting to the apical zone through an interaction with membrane-trafficking effector proteins [18]. AS-ODNs targeting *TfRABA4D* caused abnormal phenotypes in *T*. *fournieri* pollen tubes, including bulging *(*Fig. 7), similar to those observed in BFA-treated pollen tubes and *A*. *thaliana raba4d* mutants, likely due to disrupted vesicle trafficking [19]. This disruption of vesicle trafficking further leads to the loss of the gradient of pectin methylation as well as altered ROS distribution, leading to abnormal pollen tube morphology *(*Figs 2 and 7). This suggests that vesicle trafficking is critical for the deposition of cell wall materials and the ROS balance in pollen tubes and thus maintaining normal growth in *T*. *fournieri* (Fig. 8). However, it is unknown whether the initial and primary effect of vesicle-trafficking disruption is on the transport of cell wall materials, ROS distribution or the distributions of PMEs.

Additionally, the AS-ODN knockdown of *TfANX* underscores the crucial link between pollen tube integrity, rupture and ROS regulation. Under normal circumstances, rupture occurs to release the sperm cells, a process in which ROS temporarily accumulate at the tip of the pollen tube [47, 48]. However, this ROS accumulation phenomenon did not occur at the leakage site of TfANX knockdown pollen tubes, suggesting that the rupture observed in these pollens is likely due to compromised integrity (Figs 6 and 8). Furthermore, live imaging results indicate that these leakage events, which may occur multiple times, likely take place at the same shank region relative to the pollen tube tip, suggesting a potential involvement of the cross-point between de-methylesterified and methylesterified HG (Movie. S3). However, due to the lack of reliable immunostaining techniques for live pollen tubes at present, direct detection remains challenging. Nevertheless, this newly observed phenotype, which has not been reported in other studies to our knowledge, further underscores the importance of tightly regulated mechanisms that maintain pollen tube integrity during its dynamic growth process.

In flowering plants, the precise regulation of pollen tube growth is essential for successful fertilization and subsequent seed production, impacting crop yields and genetic diversity [5, 49]. Pollen tube growth is tightly controlled by a complex interplay of signaling pathways and cytoskeletal dynamics, primarily studied in model plants [5]. Here, our research on *T. fournieri*, a non-model plant, has advanced this understanding by employing chemical inhibition and AS-ODN technology to target *ANX* and *RABA4D*, which play crucial roles in pollen tube regulation [19, 41]. Our findings demonstrate that AS-ODNs can effectively suppress specific gene expression in pollen tubes, with live imaging capturing the downstream cytological dynamics in *TfANX* and *TfRABA4D* knockdown pollen tubes. This work not only contributes to the fundamental knowledge of pollen tube biology but also develops molecular techniques in reproductive tissues with agronomic value, paving a new avenue for reproductive research in non-model plants.

## Materials and methods

### Plant materials and growth conditions

*T. fournieri* plants were grown in soil at 27°C under long-day conditions (16-hour light/8-hour darkness) [29]. Flowers two days after anthesis were selected for pollen tube cultivation. Pollen was germinated and cultured in modified liquid Nitsch's medium (PGM) [50]. When germinating pollen, a dissecting needle was used to evenly disperse the pollen from the bottom anther to prevent clustering, which could lead to localized deficiencies in chemical concentrations (Figs S1C and S10). For germination rate analysis, fields of view under the microscope with similar pollen density were selected, and a five-point sampling method was employed for statistical analysis. For treatment germination, because the germination rate was altered in PGM containing BFA (1, 2, 3, and 4 μg/ml), pollen tubes grown *in vitro* were first incubated in PGM for 1 hour and the incubaed with BFA (4 μg/ml) for 2 hours (Fig. S1).

### Genome-wide identification and gene expression analysis

Seventeen *A*. *thaliana* CrRLK1L protein sequences were obtained from the TAIR website and 23 CrRLK1L proteins were identified in *T*. *fournieri* (TfCrRLK1L) based on the presence of malectin (Pfam: PF12819) and kinase (Pfam: PF07714) domains using the Pfam Protein Family database (http://pfam.xfam.org/). *RAB* genes were identified in the same way based on the *A*. *thaliana* sequence and the RAS domain (Pfam: PF00071). The expression profiles of *TfCrRLK1L* and *TfRAB* genes in different tissues and developmental stages were obtained from public transcriptome databases [51]. We used TBtools to visualize the structures of candidate genes, protein domains, and expression profiles [52]. The cDNA sequences of the genes used in this study were listed in Table S2.

### ODN selection and delivery to pollen tubes

ODNs were designed to target sequences predicted using the Sfold tool (https://sfold.wadsworth.org/cgi-bin/soligo.pl), based on the principles of nucleic acid thermostability [20]. We selected multiple 21 bp antisense fragments from the mRNA of the targeted gene, which were capped with phosphorothioate at the 5′- and 3′-ends (Table S1). These sequences had high GC contents and were synthesized by Custom Primer Invitrogen™ (Thermo, Japan).

ODNs were first diluted with 7.5 μl of jetPRIME® buffer and mixed thoroughly. Subsequently, 0.3 μl of jetPRIME® was added, and the mixture was incubated at room temperature for 10 minutes to form cationic polymers (Polyplus, France). The ODNs were then dissolved in PGM to the concentrations indicated in the text. Pollen grains were placed on this medium and incubated for 3 to 6 hours at 27°C in a humidity chamber (Fig. S10). Next, germinated pollen on PGM was stained with 0.1% Aniline blue. Three independent experiments were conducted on different days to evaluate the effects of the ODNs.

### Extraction of RNA from pollen tubes and qRT-PCR

RNA was extracted using the RNeasy Plant Mini Kit (Qiagen, USA). Pollen tubes were isolated by centrifugation at 3000 rpm. The supernatant was decanted and TRIzol was added following the manufacturer's guidelines. RNA samples were normalized to uniform concentrations using the BioSpec-nano spectrophotometer (Shimadzu, Japan). RNA was reverse-transcripted using the SuperScript II Reverse Transcriptase Kit (Invitrogen, USA), following the manufacturer's protocols. The primers listed in Table S1 were used for qRT-PCR, with *GAPDH* as the reference gene for the 2^–∆∆Ct^ technique [30].

### Cytochemical labeling and immunostaining of pollen tubes

To stain vesicles, pollen tubes were incubated with 2 μM FM4–64 in PGM for 10 minutes and washed in PGM. To stain callose, 0.1% Aniline blue was added to cultured pollen tubes. To evaluate the distribution of acidic pectin, pollen tubes were stained with 0.01% (w/v) Ruthenium red for 5 minutes and washed in PGM [5]. To assess cell membrane integrity, 2.5 μg/ml CellMask™ Plasma Membrane Stain (Invitrogen) was applied for 5 minutes followed by three rinses in PGM. To assess ROS levels, pollen tubes were incubated with 5 μM CM-H2DCFDA (Invitrogen) for 10 minutes at room temperature, followed by rinses in PGM.

Pectin localization in the pollen tube cell wall was examined using JIM5, JIM7, LM16, and LM19 antibodies [53]. Immunolabeling was conducted after fixing pollen tubes in freshly prepared 1.5% paraformaldehyde in PBS (pH 6.9) for 1.5 hours at room temperature, followed by three rinses in PBS. Pollen tubes were treated with a 1:10 dilution (in PBS) of antibodies at room temperature for 2 hours, followed by three washes in PBS. Subsequently, they were incubated with a 1:100 dilution of Alexa488-conjugated secondary antibodies at 4°C overnight and washed three times in PBS.

### Confocal microscopy and data analysis

Microscopy was conducted using the Leica STELLARIS 8. The following filters were used: for FDA, 485 nm excitation and a 515- to 525-nm emission filter; for Aniline blue, 405 nm excitation and a 450- to 490-nm bandpass emission filter; for CellMask™ Plasma Membrane Stain, 561 nm excitation and a 586- to 614-nm bandpass filter; for FM4–64, 543 nm excitation and a 560-nm long-pass emission filter; for Alexa 488, 488 nm excitation and a 515- to 565-nm emission filter; and for CM-H2DCFDA, 490 nm excitation and a 515- to 530-nm emission filter.

Confocal images were analyzed using Fiji (v. 2.14.0) and processed in Microsoft PowerPoint. A minimum of 100 independent pollen tubes were evaluated for each experiment to establish consensus phenotypes. Data are presented as means ± standard deviations (SDs). Statistical analysis was performed using Microsoft Excel software (v. 2017). Pairwise comparisons were conducted using Student's *t*-test to identify significant between-group differences. A single asterisk (*) indicates *P* < 0.05, and three asterisks (**) indicate *P* < 0.001.

## Supplementary Material

Web_Material_uhaf018

## Data Availability

The sequences of the genes used in this study were listed in Table S2 and can be accessed in the *T. fournieri* cDNA database (http://dandelion.liveholonics.com/torenia/).

## References

[1] Kanaoka MM, Higashiyama T. Peptide signaling in pollen tube guidance. Curr Opin Plant Biol. 2015;28:127–36 26580200 10.1016/j.pbi.2015.10.006

[2] Selinski J, Scheibe R. Pollen tube growth: where does the energy come from? Plant Signal Behav. 2014;9:e977200 25482752 10.4161/15592324.2014.977200 PMC4622831

[3] Ruan H, Li J, Wang T. et al. Secretory vesicles targeted to plasma membrane during pollen germination and tube growth. Front Cell Dev Biol. 2020;8:615447 33553150 10.3389/fcell.2020.615447 PMC7859277

[4] Scholz P, Anstatt J, Krawczyk HE. et al. Signalling pinpointed to the tip: the complex regulatory network that allows pollen tube growth. Plants (Basel). 2020;9:1098 32859043 10.3390/plants9091098 PMC7569787

[5] Westermann J, Srikant T, Gonzalo A. et al. Defective pollen tube tip growth induces neo-polyploid infertility. Science. 2024;383:eadh0755 38422152 10.1126/science.adh0755

[6] Guan Y, Guo J, Li H. et al. Signaling in pollen tube growth: crosstalk, feedback, and missing links. Mol Plant. 2013;6:1053–64 23873928 10.1093/mp/sst070 PMC3842152

[7] Hao GJ, Zhao XY, Zhang MM. et al. Vesicle trafficking in Arabidopsis pollen tubes. FEBS Lett. 2022;596:2231–42 35348201 10.1002/1873-3468.14343

[8] Zhou Y, Yang Y, Niu Y. et al. The tip-localized phosphatidylserine established by Arabidopsis ALA3 is crucial for Rab GTPase-mediated vesicle trafficking and pollen tube growth. Plant Cell. 2020;32:3170–87 32817253 10.1105/tpc.19.00844 PMC7534478

[9] de Graaf BH, Cheung AY, Andreyeva T. et al. Rab11 GTPase-regulated membrane trafficking is crucial for tip-focused pollen tube growth in tobacco. Plant Cell. 2005;17:2564–79 16100336 10.1105/tpc.105.033183 PMC1197435

[10] Besserer A, Burnotte E, Bienert GP. et al. Selective regulation of maize plasma membrane aquaporin trafficking and activity by the SNARE SYP121. Plant Cell. 2012;24:3463–81 22942383 10.1105/tpc.112.101758 PMC3462644

[11] Zhang L, Zhang H, Liu P. et al. Arabidopsis R-SNARE proteins VAMP721 and VAMP722 are required for cell plate formation. PLoS One. 2011;6:e26129 22022536 10.1371/journal.pone.0026129 PMC3191180

[12] Karnahl M, Park M, Mayer U. et al. ER assembly of SNARE complexes mediating formation of partitioning membrane in. elife. 2017;6:25327 10.7554/eLife.25327 PMC5438246 28525316

[13] Ma T, Li E, Li LS. et al. The Arabidopsis R-SNARE protein YKT61 is essential for gametophyte development. J Integr Plant Biol. 2021;63:676–94 32918784 10.1111/jipb.13017

[14] Ma T, Tan JR, Lu JY. et al. S-acylation of YKT61 modulates its unconventional participation in the formation of SNARE complexes in Arabidopsis. J Genet Genomics. 2024;51:1079–88 38642801 10.1016/j.jgg.2024.04.007

[15] Wang X, Xu M, Gao C. et al. The roles of endomembrane trafficking in plant abiotic stress responses. J Integr Plant Biol. 2020;62:55–69 31829507 10.1111/jipb.12895

[16] Liu L, Li C, Teo ZWN. et al. The MCTP-SNARE complex regulates Florigen transport in Arabidopsis. Plant Cell. 2019;31:2475–90 31439803 10.1105/tpc.18.00960 PMC6790074

[17] Shi Y, Luo C, Xiang Y. et al. Rab GTPases, tethers, and SNAREs work together to regulate. Front Plant Sci. 2023;14:1120841 36844074 10.3389/fpls.2023.1120841 PMC9950755

[18] Rodriguez-Furlan C, Borna R, Betz O. RAB7 GTPases as coordinators of plant endomembrane traffic. Front Plant Sci. 2023;14:1240973 37662169 10.3389/fpls.2023.1240973 PMC10470000

[19] Szumlanski AL, Nielsen E. The Rab GTPase RabA4d regulates pollen tube tip growth in Arabidopsis thaliana. Plant Cell. 2009;21:526–44 19208902 10.1105/tpc.108.060277 PMC2660625

[20] Mizuta Y, Higashiyama T. Antisense gene inhibition by phosphorothioate antisense oligonucleotide in Arabidopsis pollen tubes. Plant J. 2014;78:516–26 24495108 10.1111/tpj.12461

[21] Planchais S, Glab N, Inzé D. et al. Chemical inhibitors: a tool for plant cell cycle studies. FEBS Lett. 2000;476:78–83 10878255 10.1016/s0014-5793(00)01675-6

[22] Lam SK, Cai Y, Tse YC. et al. BFA-induced compartments from the Golgi apparatus and trans-Golgi network/early endosome are distinct in plant cells. Plant J. 2009;60:865–81 19709389 10.1111/j.1365-313X.2009.04007.x

[23] Parton RM, Fischer-Parton S, Trewavas AJ. et al. Pollen tubes exhibit regular periodic membrane trafficking events in the absence of apical extension. J Cell Sci. 2003;116:2707–19 12746485 10.1242/jcs.00468

[24] Wang Q, Kong L, Hao H. et al. Effects of brefeldin a on pollen germination and tube growth. Antagonistic effects on endocytosis and secretion. Plant Physiol. 2005;139:1692–703 16299176 10.1104/pp.105.069765 PMC1310552

[25] Kou X, Sun J, Wang P. et al. PbrRALF2-elicited reactive oxygen species signaling is mediated by the PbrCrRLK1L13-PbrMPK18 module in pear pollen tubes. Hortic Res. 2021;8:222 34608125 10.1038/s41438-021-00684-y PMC8490453

[26] Liao F, Wang L, Yang LB. et al. Antisense oligodeoxynucleotide inhibition as an alternative and convenient method for gene function analysis in pollen tubes. PLoS One. 2013;8:e59112 23527102 10.1371/journal.pone.0059112 PMC3604054

[27] Potocký M, Bezvoda R, Pejchar P. Antisense oligodeoxynucleotide-mediated gene knockdown in pollen tubes. Methods Mol Biol. 2019;1992:359–65 31148051 10.1007/978-1-4939-9469-4_24

[28] Susaki D, Takeuchi H, Tsutsui H. et al. Live imaging and laser disruption reveal the dynamics and cell-cell communication during *Torenia fournieri* female gametophyte development. Plant Cell Physiol. 2015;56:1031–41 25713175 10.1093/pcp/pcv031

[29] Higashiyama T, Inatsugi R, Sakamoto S. et al. Species preferentiality of the pollen tube attractant derived from the synergid cell of *Torenia fournieri*. Plant Physiol. 2006;142:481–91 16935992 10.1104/pp.106.083832 PMC1586061

[30] Okuda S, Tsutsui H, Shiina K. et al. Defensin-like polypeptide LUREs are pollen tube attractants secreted from synergid cells. Nature. 2009;458:357–61 19295610 10.1038/nature07882

[31] Mizukami AG, Inatsugi R, Jiao J. et al. The AMOR arabinogalactan sugar chain induces pollen-tube competency to respond to ovular guidance. Curr Biol. 2016;26:1091–7 27068416 10.1016/j.cub.2016.02.040

[32] Okuda S, Suzuki T, Kanaoka MM. et al. Acquisition of LURE-binding activity at the pollen tube tip of *Torenia fournieri*. Mol Plant. 2013;6:1074–90 23482369 10.1093/mp/sst050

[33] Yanagisawa N, Kozgunova E, Grossmann G. et al. Microfluidics-based bioassays and imaging of plant cells. Plant Cell Physiol. 2021;62:1239–50 34027549 10.1093/pcp/pcab067 PMC8579190

[34] Grebnev G, Cvitkovic M, Fritz C. et al. Quantitative structural organization of bulk apical membrane traffic in pollen tubes. Plant Physiol. 2020;183:1559–85 32482906 10.1104/pp.20.00380 PMC7401101

[35] Gebert M, Dresselhaus T, Sprunck S. F-actin organization and pollen tube tip growth in *Arabidopsis* are dependent on the gametophyte-specific armadillo repeat protein ARO1. Plant Cell. 2008;20:2798–814 18931021 10.1105/tpc.108.061028 PMC2590741

[36] Li H, Yang Y, Zhang H. et al. The *Arabidopsis* GPI-anchored protein COBL11 is necessary for regulating pollen tube integrity. Cell Rep. 2023;42:113353 38007687 10.1016/j.celrep.2023.113353

[37] Chebli Y, Kaneda M, Zerzour R. et al. The cell wall of the *Arabidopsis* pollen tube—spatial distribution, recycling, and network formation of polysaccharides. Plant Physiol. 2012;160:1940–55 23037507 10.1104/pp.112.199729 PMC3510122

[38] Xiong J, Yang Y, Fu G. et al. Novel roles of hydrogen peroxide (H₂O₂) in regulating pectin synthesis and demethylesterification in the cell wall of rice (Oryza sativa) root tips. New Phytol. 2015;206:118–26 25615266 10.1111/nph.13285

[39] Do , Choi H, Palmgren M. et al. ABCG28 is required for the apical accumulation of reactive oxygen species in growing pollen tubes. Proc Natl Acad Sci USA. 2019;116:12540–9 31152136 10.1073/pnas.1902010116 PMC6589667

[40] De Smedt SC, Demeester J, Hennink WE. Cationic polymer based gene delivery systems. Pharm Res. 2000;17:113–26 10751024 10.1023/a:1007548826495

[41] Boisson-Dernier A, Roy S, Kritsas K. et al. Disruption of the pollen-expressed FERONIA homologs ANXUR1 and ANXUR2 triggers pollen tube discharge. Development. 2009;136:3279–88 19736323 10.1242/dev.040071 PMC2739144

[42] Sugi N, Susaki D, Mizuta Y. et al. Letter to the editor: blue light irradiation induces pollen tube rupture in various flowering plants. Plant Cell Physiol. 2024;65:704–7 38466564 10.1093/pcp/pcae018 PMC11138365

[43] Kim YJ, Kim MH, Hong WJ. et al. OsMTD2-mediated reactive oxygen species (ROS) balance is essential for intact pollen-tube elongation in rice. Plant J. 2021;107:1131–47 34143922 10.1111/tpj.15373

[44] Lassig R, Gutermuth T, Bey TD. et al. Pollen tube NAD(P)H oxidases act as a speed control to dampen growth rate oscillations during polarized cell growth. Plant J. 2014;78:94–106 24506280 10.1111/tpj.12452

[45] Higashiyama T, Takeuchi H. The mechanism and key molecules involved in pollen tube guidance. Annu Rev Plant Biol. 2015;66:393–413 25621518 10.1146/annurev-arplant-043014-115635

[46] Bost JP, Barriga H, Holme MN. et al. Delivery of oligonucleotide therapeutics: chemical modifications, lipid nanoparticles, and extracellular vesicles. ACS Nano. 2021;15:13993–4021 34505766 10.1021/acsnano.1c05099 PMC8482762

[47] Duan Q, Kita D, Johnson EA. et al. Reactive oxygen species mediate pollen tube rupture to release sperm for fertilization in Arabidopsis. Nat Commun. 2014;5:3129 24451849 10.1038/ncomms4129

[48] Ge Z, Cheung AY, Qu LJ. Pollen tube integrity regulation in flowering plants: insights from molecular assemblies on the pollen tube surface. New Phytol. 2019;222:687–93 30556141 10.1111/nph.15645

[49] Huang J, Dong J, Qu LJ. From birth to function: male gametophyte development in flowering plants. Curr Opin Plant Biol. 2021;63:102118 34625367 10.1016/j.pbi.2021.102118 PMC9039994

[50] Higashiyama T, Kuroiwa H, Kawano S. et al. Guidance in vitro of the pollen tube to the naked embryo sac of *Torenia fournieri*. Plant Cell. 1998;10:2019–31 9836742 10.1105/tpc.10.12.2019 PMC143976

[51] Morinaka H, Mamiya A, Tamaki H. et al. Transcriptome dynamics of epidermal reprogramming during direct shoot regeneration in *Torenia fournieri*. Plant Cell Physiol. 2021;62:1335–54 34223624 10.1093/pcp/pcab101 PMC8579340

[52] Chen C, Wu Y, Li J. et al. TBtools-II: a "one for all, all for one" bioinformatics platform for biological big-data mining. Mol Plant. 2023;16:1733–42 37740491 10.1016/j.molp.2023.09.010

[53] Bidhendi AJ, Chebli Y, Geitmann A. Fluorescence visualization of cellulose and pectin in the primary plant cell wall. J Microsc. 2020;278:164–81 32270489 10.1111/jmi.12895

