# Supplementary material for: Investigating vesicle-mediated regulation of pollen tube growth through BFA inhibition and AS-ODN targeting of TfRABA4D in Torenia fournieri
Source: Hortic Res. 2025 Jan 15;12(4):uhaf018. doi: 10.1093/hr/uhaf018 (PMC11908828; doi:10.1093/hr/uhaf018)
Supplement: Web_Material_uhaf018 [file web_material_uhaf018.zip › SupplementaryFigure.pdf]

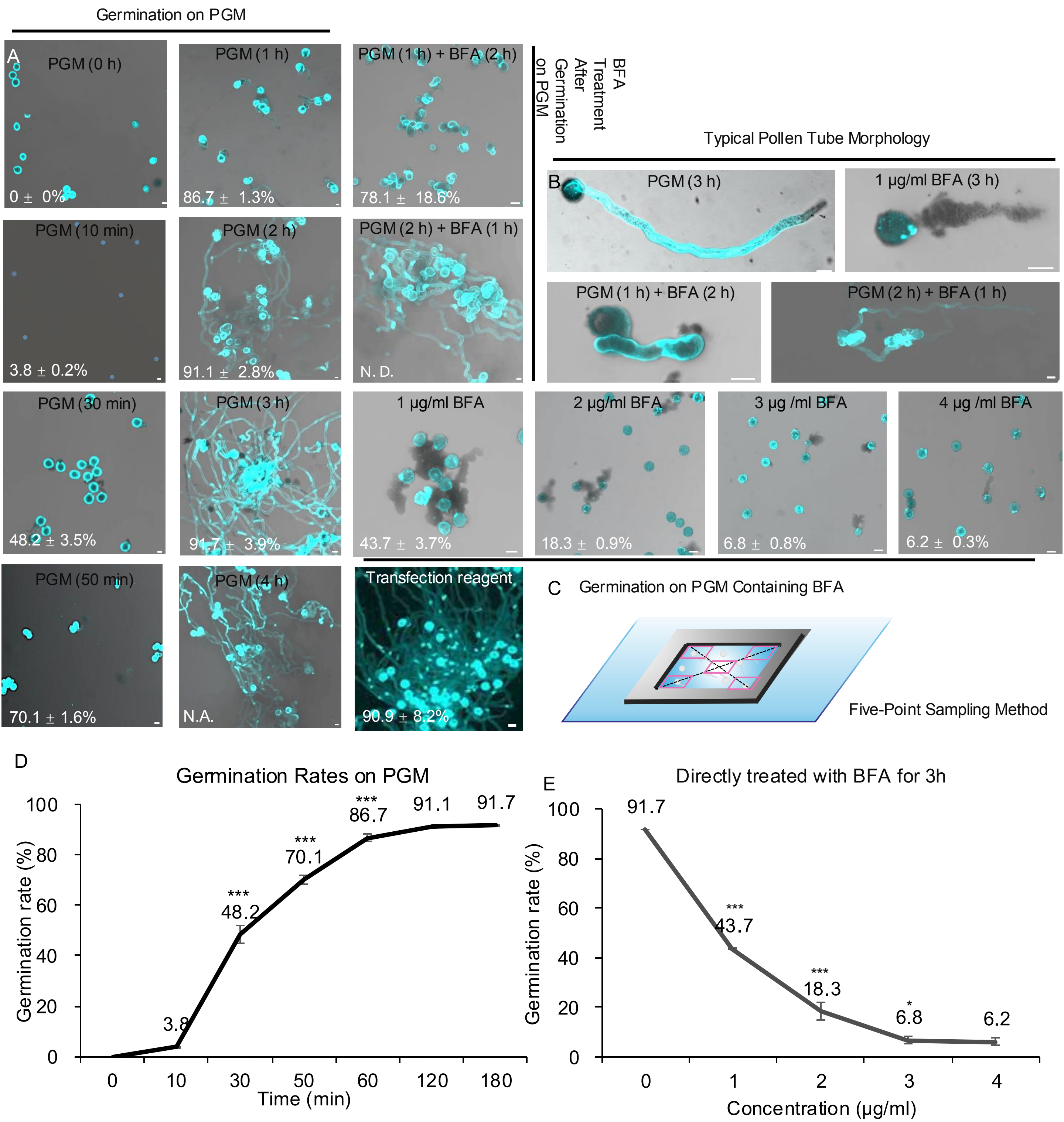

Fig. S1. Effects of chemical treatment at the indicated concentrations on pollen germination and pollen tube growth

A *T. fournieri* pollen tubes cultured in PGM for 0 h, 10 min, 30 min, 50 min, 1 h, 2 h, 3 h and 4 h; PGM for 1h followed by 4 μg/ml BFA for 2 h; PGM for 1h followed by 4 μg/ml BFA for 2 h; directly cultured in PGM containing 1 μg/ml BFA, 2 μg/ml BFA, 3 μg/ml BFA and 4 μg/ml BFA; cultured in PGM containing transfection reagent. " N.A. " indicates "not applicable", due to high pollen tube density at 4 hours, making germination rate calculation impossible. " N.D. " indicates "not determined", due to unstable phenotypes in the Blank 2h + BFA 1h treatment. Scale bars: 10 μm. **B** Typical morphology of pollen tubes cultured in PGM for 3 h, 1 μg/ml BFA for 3 h, in PGM for 1 h followed by 4 μg/ml BFA for 2 h and in PGM for 2 h followed by 4 μg/ml BFA for 1 h. The cyan color of the pollen tubes in panels was stained with Aniline blue. **C** Schematic representation of the five-point sampling method for calculating germination rate. **D** Line graph showing the variation in pollen germination rate over time in PGM. **E** Line graph showing the germination rate of pollen in PGM with varying concentrations of BFA. Values are means and standard deviations of more than 3 replicate experiments (n > 200).

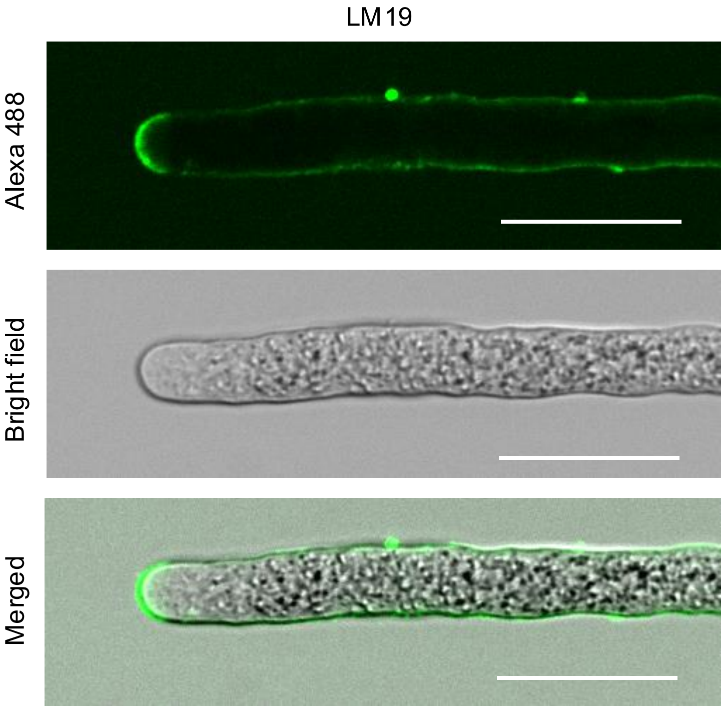

Fig. S2. Immunofluorescent labeling of de-methylesterified homogalacturonan in pollen tubes using LM19 monoclonal antibody

The image set includes fluorescence, bright-field, and merged views. Scale bars: 20 μm. Assays had three biological replicates, and representative figures were shown.

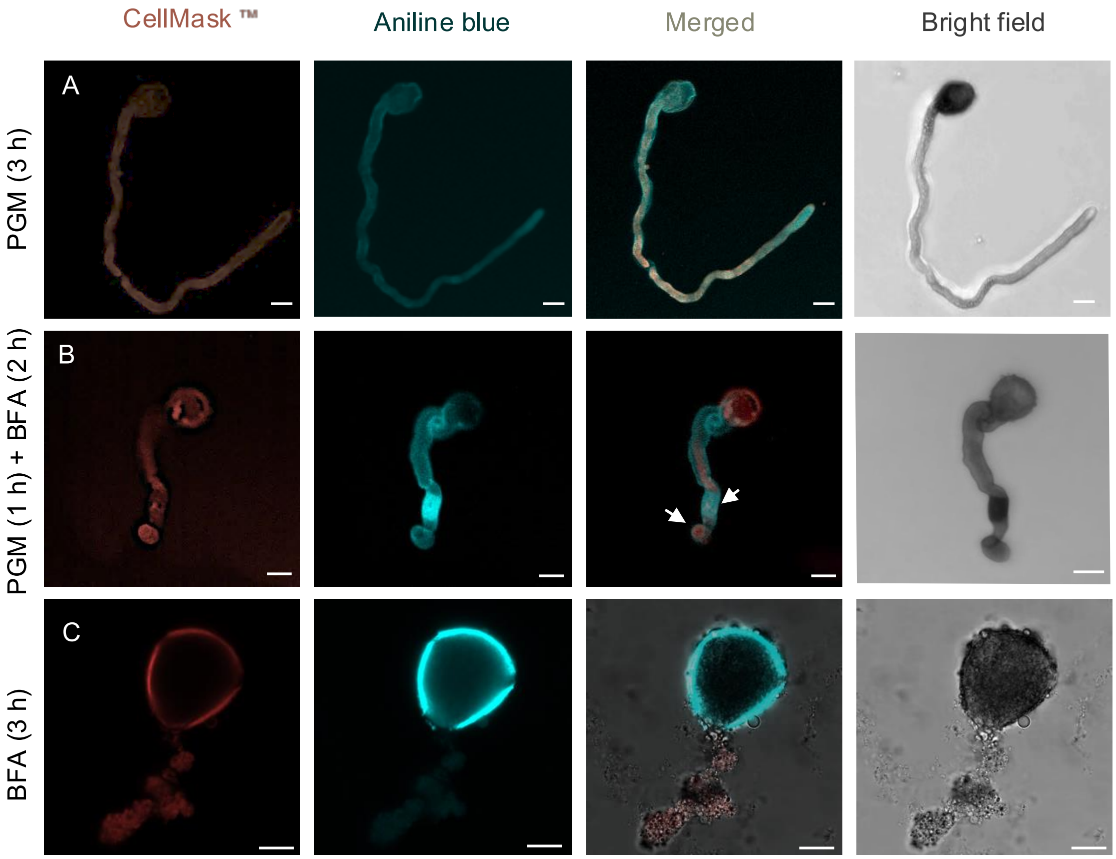

Fig. S3. Cytochemical labeling of cell membrane and cell wall components

A The image set of a pollen tube cultured in PGM (3 h) stained with CellMask™ and Aniline blue, includes CellMask™, Aniline blue, merged, and bright-field views. Scale bars: 10 µm. **B** The image set of a pollen tube cultivated in PGM (1 h) and BFA (2 h) stained with CellMask™ and Aniline blue, includes CellMask™, Aniline blue, merged, and bright-field views. White arrows indicate the abnormal callose distribution after PGM (1 h) and BFA (2 h) treatment. Scale bars: 10 µm. **C** The image set of a pollen tube cultured in BFA (3 h) stained with CellMask™ and Aniline blue, includes CellMask™, Aniline blue, merged and bright-field views. Scale bars: 10 µm. Each treatment had three biological replicates, and representative figures were shown.

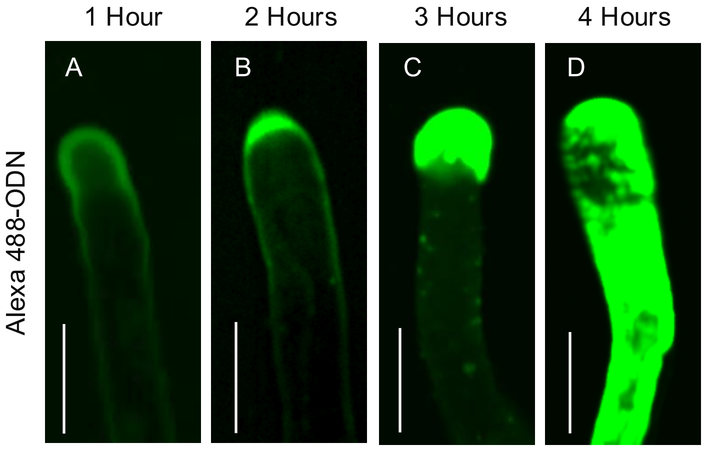

Fig. S4. Uptake of antisense ODNs into pollen tubes cultivated in PGM for 4 h

The distribution of Alexa 488-labeled ODN in pollen tubes after 1 (**A**), 2 (**B**), 3 (**C**), and 4 (**D**) hours of incubation. Scale bars: 10 µm. Assays had three biological replicates, and representative figures were shown.

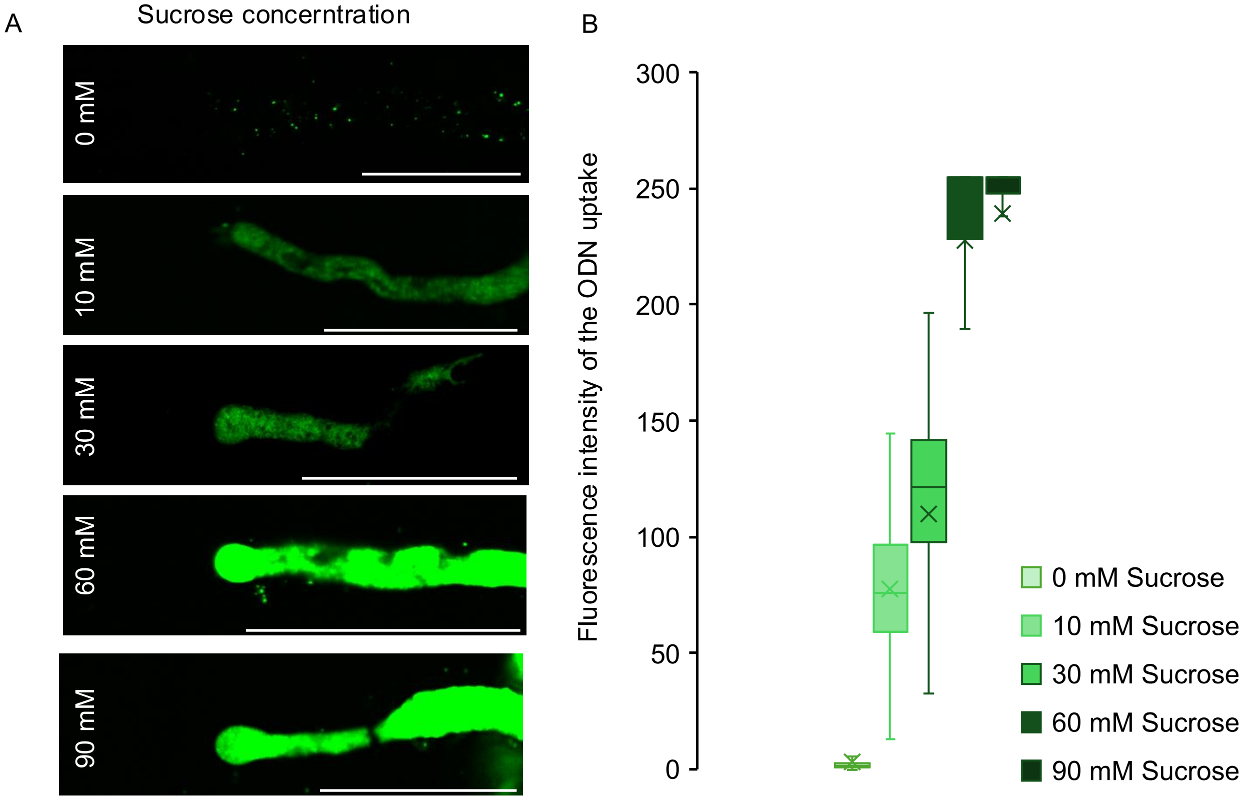

Fig. S5. Uptake of ODNs by pollen tubes after 3 hours of incubation in PGM with varying concentrations of sucrose
A The distribution of Alexa 488-labeled ODN in pollen tubes after 3 hours of incubation in PGM containing 0 mM, 10 mM, 30 mM, 60 mM, and 90 mM sucrose. Scale bars: 50 µm. **B** Box plot showing the fluorescence intensity of Alexa 488-labeled ODN distributed in pollen tubes. Values are from 3 replicate experiments ($n \geq 5$).

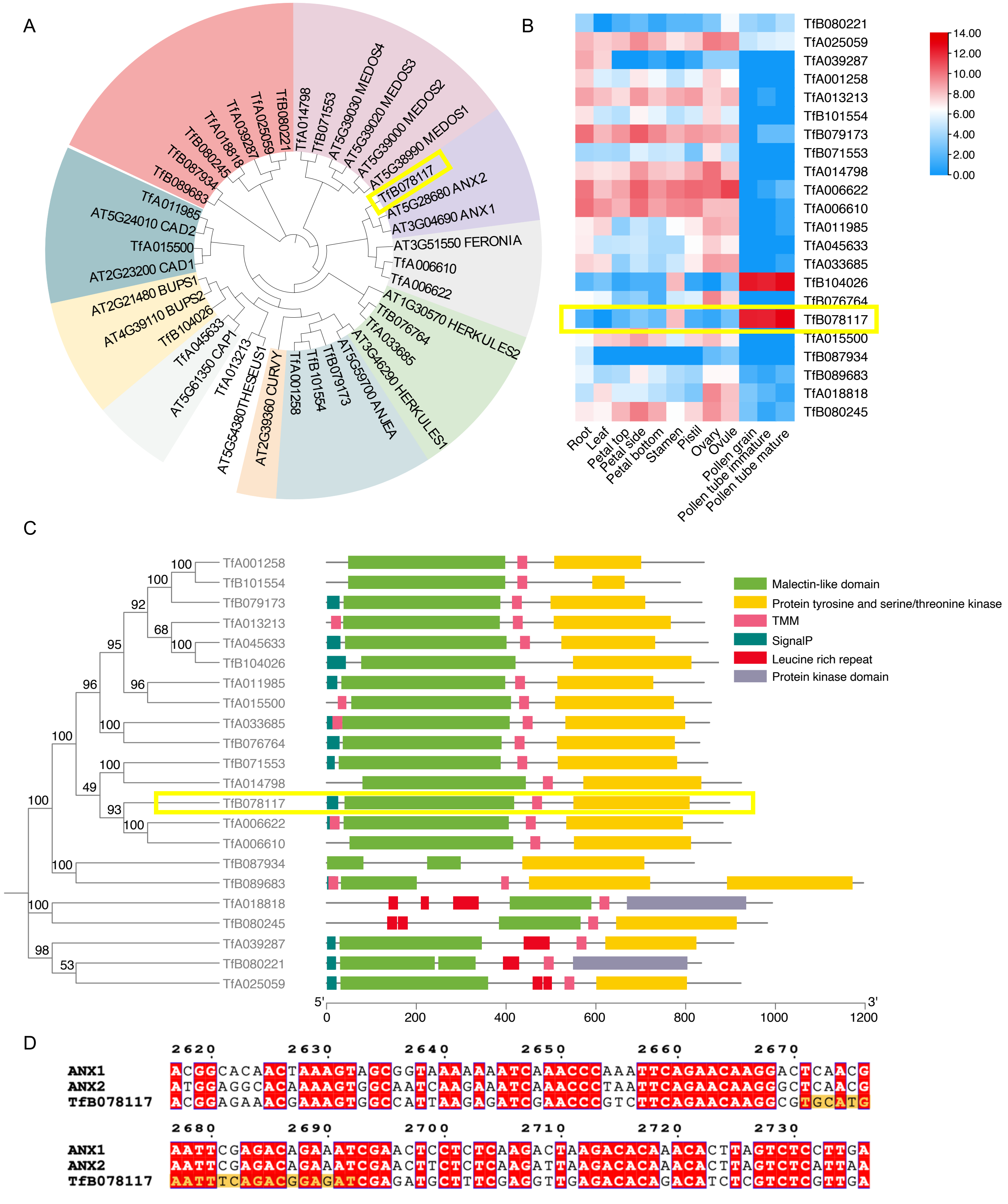

Fig. S6. Genome-wide investigation of the *CrRLK1L* gene family and its expression profile in *T. fournieri*

A Evolutionary relationships of CrRLK1Ls in *A. thaliana* and *T. fournieri*. Seventeen *A. thaliana* CrRLK1L protein sequences were obtained from the TAIR website and 22 CrRLK1L proteins were identified in *T. fournieri*; these had malectin (pfam: TfB080671) and kinase (pfam: PF07714) domains. **B** Expression profile of *TfCrRLK1L* family members. **C** Domain structure of *TfCrRLK1L* family members. TfB078117, which had the closest evolutionary relationship to *ANX1* and *ANX2*, is highly expressed in pollen tubes and has a complete signal peptide and transmembrane region. Consequently, it is considered a homolog of *ANX* in *T. fournieri* and designated as *TfANX*. Yellow square marks *TfB078117*. **D** Alignments of *TfB078117* with *FERONIA*, *ANX1,* and *ANX2*, the ODN design site is highlighted in yellow.

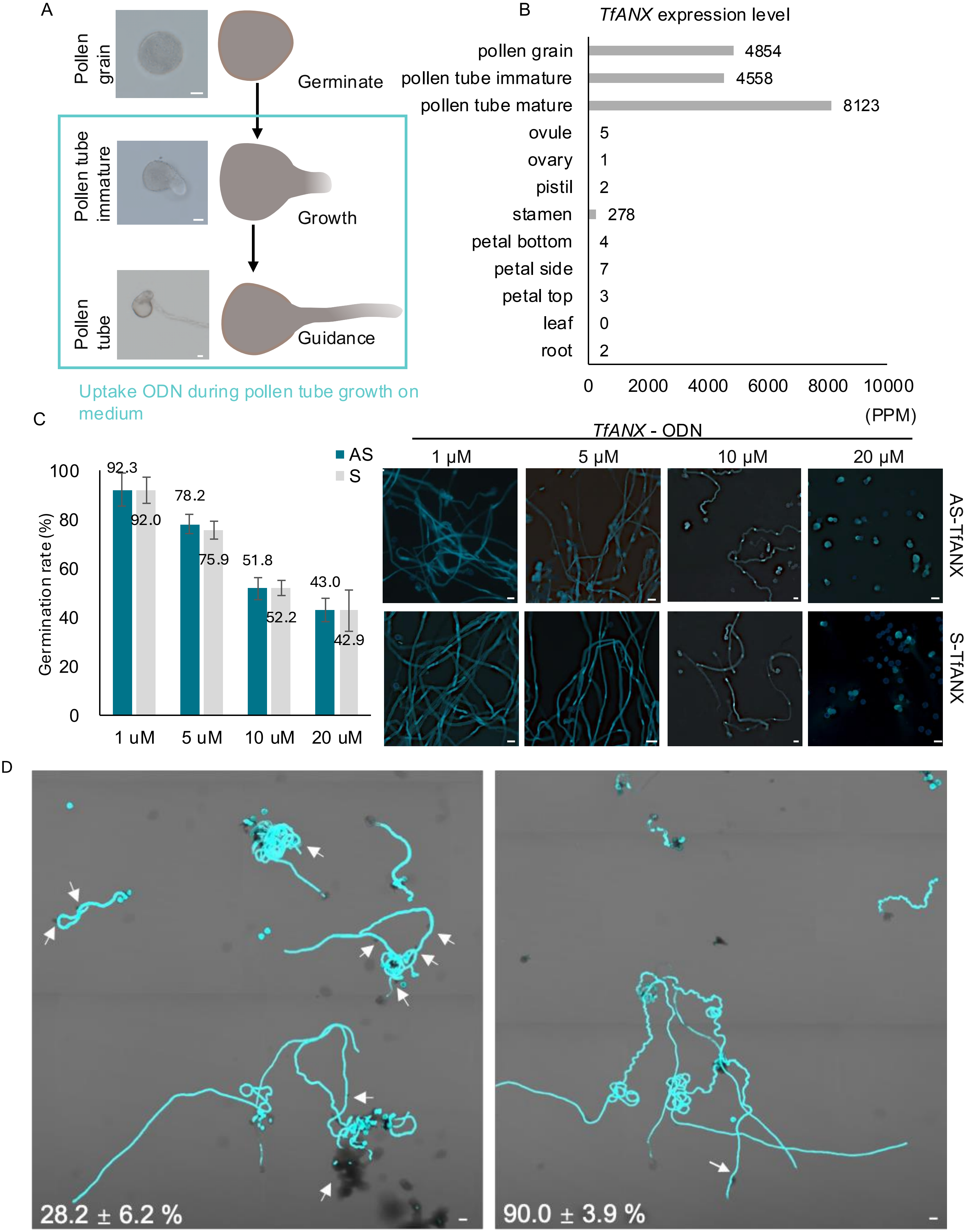

Fig. S7. *TfANX* gene expression profile and concentration dependence

A Diagram of pollen tube germination. **B** Expression profile of the *TfANX* gene in various tissues. The blue box represents the developmental period possible on PGM medium with ODN. The immature and mature states of pollen tubes are distinguished by their ability to respond to female attractants, as noted in reference 44. **C** Germination of pollen grains incubated for 3 h was examined in PGM containing the indicated concentrations of AS- and S-ODN. Scale bars: 10 μm. Concentration-dependent effects of ODNs on the pollen germination rate (n > 200). Arrows indicate abnormally developed pollen tubes. *** $P < 0.001$ from the control. **D** Panoramic view of pollen tubes under 10 μM AS-TfANX treatment and S-TfANX treatment. Scale bars: 10 μm. The white arrow indicates the site of leakage. Each treatment had three biological replicates.

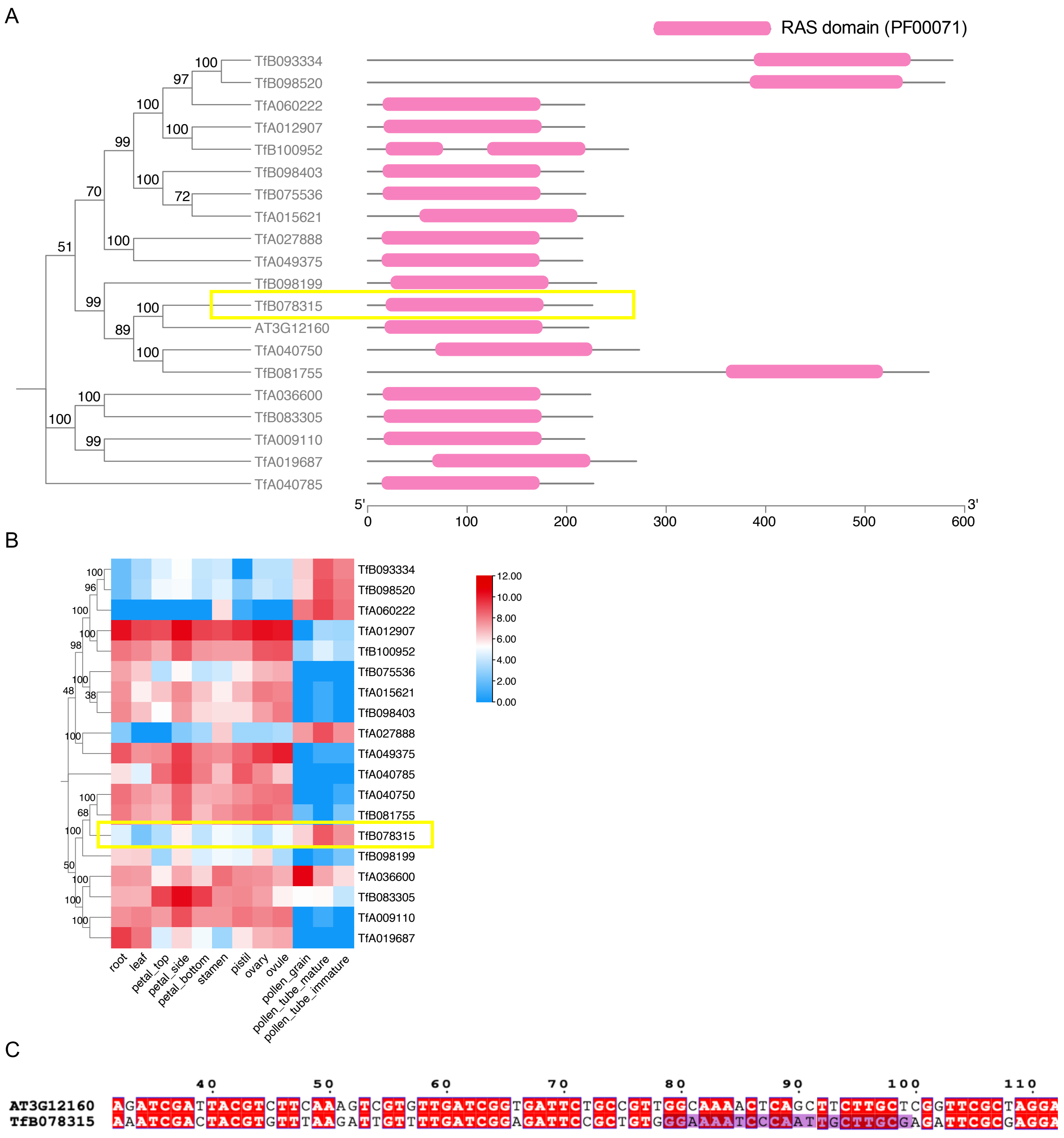

Fig. S8. Genome-wide investigation of *RABA4D* homologs and their expression profiles in *T. fournieri*

A Evolutionary relationship of RABA4D and their domain structure in *A. thaliana* and *T. fournieri*. *A. thaliana* RABA4D protein sequences were obtained from the TAIR website; 19 RABA4D homologs had a RAS domain (pfam: PF00071). **B** Expression profile of *RABA4D* homologs. Yellow square indicates the location of *TfB078315*. **C** Alignment of *TfB078315* with *RABA4D*; purple highlight indicates ODN site. *TfB078315*, which had the closest evolutionary relationship to *RABA4D*, is highly expressed in mature pollen. Consequently, it was considered a homolog of *RABA4D* in *T. fournieri* and was designated *TfRABA4D*.

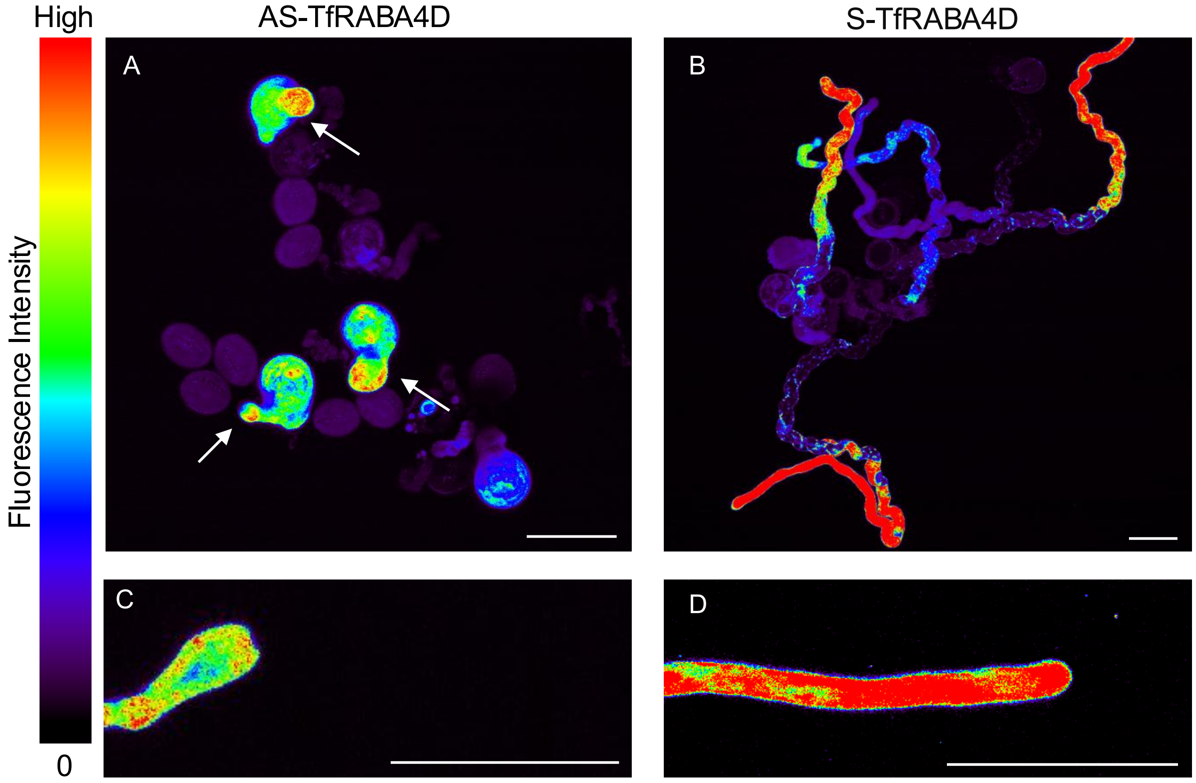

Fig. S9. ROS distribution in pollen tube tips of *T. fournieri* under AS-TfRABA4D and S-TfRABA4D treatment

A, B. Images ROS distribution of pollen grains germinated in 10 µM AS-TfRABA4D (**A**) and 10 µM S-TfRABA4D (**B**) medium. Scale bars: 50 µm. Arrows indicate abnormally enlarged pollen tubes. **C, D**. Representative images of ROS distribution in the pollen tube tips of **A** and **B**. Scale bars: 50 µm. The ROS distribution was detected with CM-H2DCFDA.

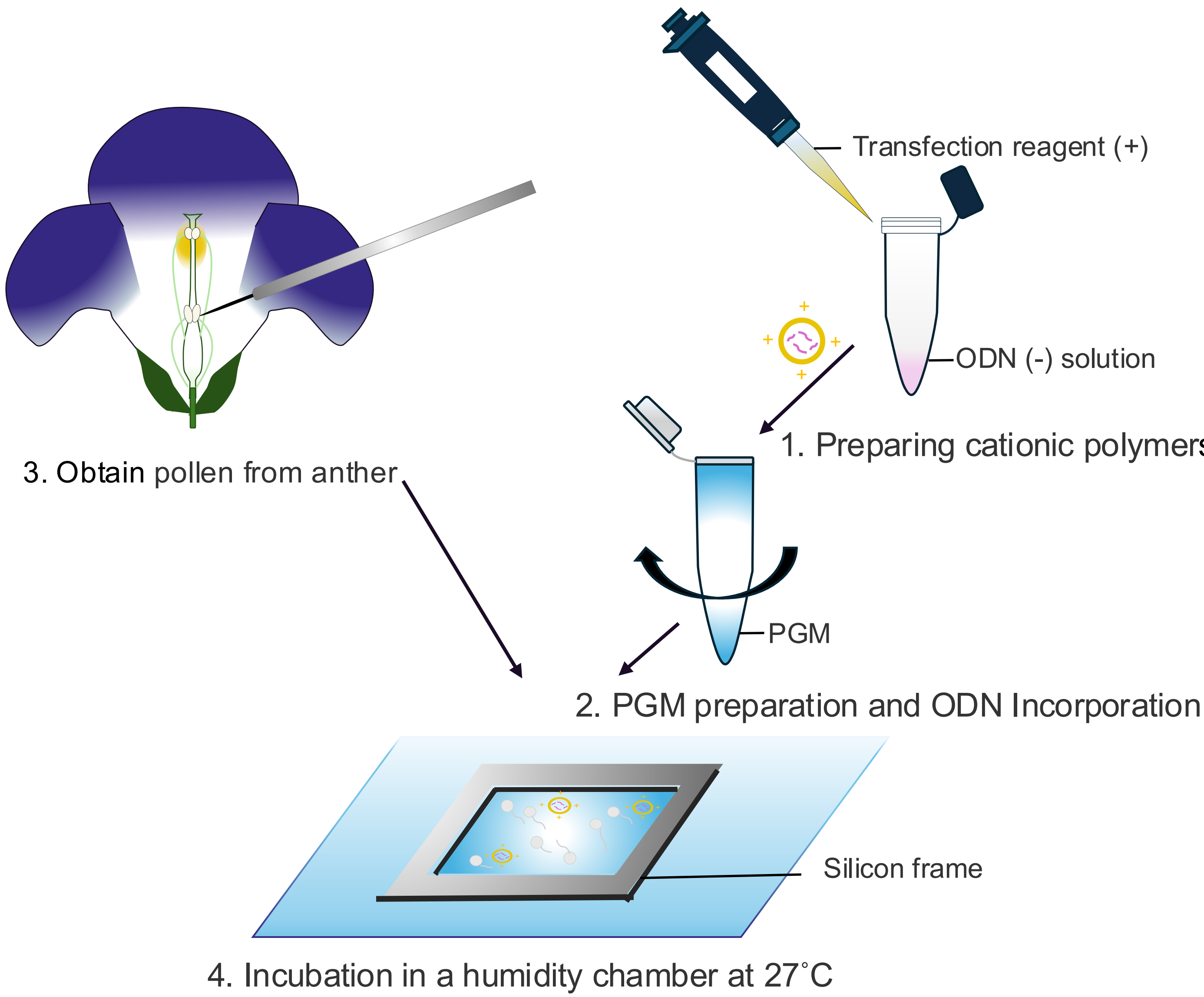

Fig. S10. Schematic representation of ODN-mediated inhibition in pollen tube of *T. fournieri*
